# Animal models for filovirus infections

**DOI:** 10.24272/j.issn.2095-8137.2017.053

**Published:** 2018-02-09

**Authors:** Vinayakumar Siragam, Gary Wong, Xiang-Guo Qiu

**Affiliations:** 1 Special Pathogens Program, Public Health Agency of Canada, Winnipeg, Manitoba R3E 3R2, Canada; 2 Department of Medical Microbiology, University of Manitoba, Winnipeg, Manitoba R3E 0J9, Canada; 3 Shenzhen Key Laboratory of Pathogen and Immunity, State Key Discipline of Infectious Disease, Shenzhen Third People’s Hospital, Shenzhen Guangzhou 518020, China; 4 Key Laboratory of Pathogenic Microbiology and Immunology, Institute of Microbiology, Chinese Academy of Sciences, Beijing 100101, China

**Keywords:** Filovirus, Ebola virus, Marburg virus, Marburg virus disease, Ebola virus disease, Mouse-adapted ebolavirus, Guinea-pig-adapted ebolavirus, Sudan virus, Plaque-forming units

## Abstract

The family *Filoviridae*, which includes the genera *Marburgvirus* and *Ebolavirus*, contains some of the most pathogenic viruses in humans and non-human primates (NHPs), causing severe hemorrhagic fevers with high fatality rates. Small animal models against filoviruses using mice, guinea pigs, hamsters, and ferrets have been developed with the goal of screening candidate vaccines and antivirals, before testing in the gold standard NHP models. In this review, we summarize the different animal models used to understand filovirus pathogenesis, and discuss the advantages and disadvantages of each model with respect to filovirus disease research.

## INTRODUCTION

The family *Filoviridae* consists of three genera, *Ebolavirus*, *Marburgvirus*, and *Cuevavirus* (Kuhn et al., 2011, 2013). Filoviruses are classified as biosafety level 4 (BSL-4) pathogens and infections result in severe hemorrhagic fevers in humans and non-human primates (NHPs), with fatality rates as high as 90% (Sanchez et al., 2007). *Marburgvirus* has two member viruses: Marburg virus (MARV) and Ravn virus (RAVV). *Ebolavirus* has five member viruses: Ebola virus (EBOV), Sudan virus (SUDV), Tai Forest virus (TAFV), Bundibugyo virus (BDBV), and Reston virus (RESTV) (Kuhn et al., 2013). Live viruses belonging to *Cuevavirus* have not yet been isolated. Currently, no approved vaccines or therapeutics are available against filovirus infections; however, clinical trials of candidate vaccines and therapeutics conducted during the 2014–2016 EBOV outbreak in West Africa (Martin et al., 2016; Wong & Kobinger, 2015) provide hope that a licensed medical countermeasure is on the horizon.

The transmission of filoviruses to humans is poorly understood, but outbreaks are likely started by direct contact with dead animal carcasses (typically NHPs) or in the case of MARV, the bat reservoir, resulting in subsequent transmission within the susceptible human population (Leroy, 2009). The blood and/or other body fluids of dead animals and humans are especially infectious, with viremia in human patients reaching 10^8^ copies/mL of blood (Faye et al., 2015). Contact with blood and bodily fluids is thought to be the main mode of transmission. After an incubation period of 2–21 days, general symptoms such as fever, chills, fatigue, headache, and myalgia appear. As disease progresses, systemic (prostration, lethargy), gastrointestinal (anorexia, vomiting, abdominal pain, diarrhea), respiratory (chest pain, breath shortness, cough, nasal discharge), vascular (conjunctival injection, postural hypotension, edema), and neurological (headache, confusion, coma) symptoms may appear, sometimes accompanied with hemorrhage from venipuncture sites. In fatal cases, patients succumb after multiple organ failure between 6–16 days after the onset of symptoms (Nakayama & Saijo, 2013).

Animal models against filoviruses have been developed in mice, guinea pigs, hamsters, ferrets and NHPs (Bente et al., 2009; Bradfute et al., 2012; Connolly et al., 1999; Marzi et al., 2016; Kozak et al., 2016; Kroeker et al., 2017b; Yamaoka et al., 2017). The development of animal models that recapitulates hallmarks of human filoviral disease is crucial in understanding the pathogenesis of these viruses. In this review, we summarize the animal models that have been developed for filoviruses (Table 1), and discuss the advantages and disadvantages of each animal model (Table 2). 

**Table 1:** Animal models for studying filovirus infections

Filovirus species	Immuno-competent mouse	Immuno-compromised mouse	Guinea pig	Syrian hamster	Ferret	NHP
EBOV	+	+	+	+	+	+
MARV	+	+	+	+	-	+
RAVV	+	+	+	-	-	+
SUDV	-	+	+	-	+	+
TAFV	-	-	-	-	-	-
BDBV	-	-	-	-	+	+

NHP: Non-human primate; EBOV: Ebola virus; MARV: Marburg virus; RAVV: Ravn virus; SUDV: Sudan virus; TAFV: Tai Forest virus; BDBV: Bundibugyo virus; “+”: Available; “-”: Not available.

**Table 2:** Advantages and disadvantages of animal models for filovirus infections

Animal models	Advantages	Disadvantages
Mice	Low cost, easy to use Transgenic and knockout models are available	Only i.p. infection is 100% lethal Mouse-adapted variants needed
Guinea pigs	Low cost, larger animals to study disease progression and easy to use	Transgenic and knockout models are not available Lack of immunological tools and reagents to evaluate cell-mediated responses to vaccines and therapeutics Guinea pig-adapted variants needed
Syrian Hamsters	An alternative to guinea pigs Elegant model to compare differences in immune responses to EBOV infection	Mouse-adapted variants needed Lack of commercially available reagents and host genomic information Transgenic and knockout models are available
Ferrets	Small body size, low cost Closely resembles to human filoviral disease Can use clinical isolates to study pathogenesis	Limited availability of ferret-specific reagents Ferret immune responses are poorly understood Transgenic and knockout models are available
NHPs	Gold standard model to evaluate filovirus infections and closely recapitulate human disease	Animals are expensive, ethical considerations and extensively husbandry requirements needed Transgenic and knockout models are not available

NHPs: Non-human primates.

## MOUSE MODELS

Mice are easy to handle in the laboratory, are available commercially in large numbers, and have a low unit cost. Due to the wide availability of biochemical reagents and immunological tools, mice are preferred as the small animal model for filovirus research (Bradfute et al., 2007).

### Immunocompetent Mouse Model

#### Ebola virus (EBOV)

As immunocompetent mice are resistant to infections with wild type EBOV (WT-EBOV) (Banadyga et al., 2016; Bray, 2001; Warfield et al., 2009), Bray et al. (1998) adapted the 1976 Mayinga isolate of EBOV to cause lethal disease in adult immunocompetent mice through the sequential passaging of infected liver homogenates in newborn mice. The resultant plaque-purified mouse-adapted EBOV (MA-EBOV) was lethal for adult BALB/c, CD-1, and C57BL/6 mice at 30 or 3 000 median lethal dose (LD_50_) of 10^0^ or 10^2^ plaque-forming units (pfu) when injected intraperitoneally (i.p.). Remarkably, infection with MA-EBOV did not produce any disease symptoms via the subcutaneous (s.c.) or intramuscular (i.m.) routes at doses of 10^6^ pfu (Bray et al., 1998). Compared with WT-EBOV, MA-EBOV exhibited eight amino acid changes in the coding and non-coding regions of the virus genome (Ebihara et al., 2006), with amino acid changes observed in viral protein (VP) 35, VP24, nucleoprotein (NP), and RNA-dependent RNA polymerase (L) genes. Furthermore, the determinants of virulence in mice were shown to be mutations in NP and VP24, in which recombinant viruses containing only the NP and VP24 mutations were resistant to type I interferon (IFN) *in vitro* and conferred lethality to mice (Ebihara et al., 2006). The disease observed in adult immunocompetent mice by i.p. inoculation of MA-EBOV closely reflected EBOV infection in humans, and the virus replicated well in blood, reaching peak titers of10^9^ pfu/mL. Biochemical and histopathological studies have shown diminished functions in the liver and kidney (Gibb et al., 2001b). In addition, lymphocyte apoptosis and decrease in platelet counts were observed in mice infected with MA-EBOV. In livers of MA-EBOV-infected mice, viral replication was seen in hepatocytes, Kupffer cells, and sinusoidal endothelial lining cells. In the spleen, viral antigen was noted at day 2 after infection. Coagulopathy, such as disseminated intravascular coagulation with prolongation of prothrombin time (PT) and activated partial thromboplastin time (APTT) have also been detected at advanced stages of the disease (Bray et al., 2001; Warfield et al., 2009). However, fibrin deposition and breakdown at sites of viral replication in the spleen and other tissues have not been observed in tissue sections of MA-EBOV-infected mice (Bray et al., 1998). The MA-EBOV initially targets macrophages and other mononuclear phagocytes to invade the regional lymph nodes, similar to observations in humans, NHPs, and guinea pigs (Connolly et al., 1999; Davis et al., 1997; Gibb et al., 2001a, b; Zaki & Goldsmith, 1999). Thus, immunocompetent mouse models are considered ideal for studying fundamental aspects of viral replication, pathogenesis, and counter immune responses. They also provide an excellent platform for evaluating the therapeutic efficacy of candidate vaccines, antibodies, and small molecules such as anti-viral drugs (Kroeker et al., 2017a; Mendoza et al., 2016; Qiu et al., 2016; Zeitlin et al., 2016).

#### Marburg virus (MARV)

Marburg virus (MARV) and RAVV variants lethal to adult immunocompetent mice have been developed through sequential passage in the livers and spleens of severe, combined immunodeficient (SCID) mice (Warfield et al., 2007). Qiu et al. (2014) established a lethal mouse model against the Angolan strain MARV (MARV/Ang), the most virulent strain of MARV, with mice infected with 2 000 LD_50_ of MARV/Ang-MA i.p. showing lymphopenia, thrombocytopenia, and uncontrolled viremia. Warfield et al. (2009) developed a lethal mouse-adapted variant of RAVV (MA-RAVV), which caused death 5–10 days post infection (dpi), with disease manifestations including high viremia, fibrin degradation products, platelet loss, profound loss of tissue lymphocytes, and liver damage 

### Immunocompromised Mouse Model

#### Ebola virus (EBOV)

Another method of studying WT filovirus infections in mice involves the use of mice with defects in the immune system. Bray et al. (2001) reported that i.p. inoculation of WT-EBOV was lethal to IFN-α/β receptor knockout (IFNAR^–/–^) mice, with immunohistochemical analysis demonstrating increased replicating virus and viral antigen in livers and spleens. Additionally, mice knocked out for transcription factor STAT1 (which plays a key role in type I IFN signaling) were also highly susceptible to i.p. inoculation with 10^2^ pfu of WT-EBOV (Bray et al., 2001). 

The type I IFN response plays a key role to resistance in mice. In addition, mouse models deficient in type I IFN signaling are more susceptible to WT filoviruses, serving an important model for studying pathogenesis. Immunodeficient mouse models of EBOV infection demonstrate high viremia and viral load in the spleen, liver, and multiple organ tissues. Lymphopenia, thrombocytopenia, kidney dysfunction, and liver damage have been observed in disease progression (Bradfute et al., 2007; Bradfute et al., 2010; Bray et al., 2001; Shurtleff & Bavari, 2015). As immunocompromised mice have a higher unit cost compared with immunocompetent mice, require handling in sterile conditions, and cannot mount a normal robust immune response (thus cannot determine immune correlates of protection or pathogenesis), they have not been used as extensively for research. However, the advantage to this animal model is that clinical WT filovirus strains can be used as a challenge virus to test the efficacy of candidate countermeasures rapidly without the need for host adaptation, which can be time consuming.

#### Marburg virus (MARV)

Warfield et al. (2007) examined SCID mice (which lack B and T cells) with intact IFN responses, who became sick when inoculated i.p. with ~10^3^ pfu of ‘SCID mouse-adapted’ MARV-Musoke and died within 3–4 weeks after infection. However, after subsequent animal-to-animal passages, both RAVV and Musoke strains of MARV (MARV-Musoke and MARV-Ci67) caused rapidly lethal disease (Warfield et al., 2007). Initial signs of MARV disease in these animal models include fever, anorexia, rash, huddling, weight loss, dehydration, and diarrhea. Severe complications such as prostration, failure to respond to stimulation, hind limb paralysis, and bleeding from body orifices develop at 6–10 days. Early hematological and immunological changes include lymphopenia, neutrophilia, and profound thrombocytopenia, whereas alterations in serum chemistry levels, such as increases in liver enzymes, are also prominent after infection. At death, MARV was found at high titers in the blood, liver, spleen, kidneys, and other major organs, indicating systemic spread of the virus (Warfield et al., 2007).

#### Sudan virus (SUDV)

Mice deficient for type 1 IFN infections (α/β/γ IFN receptor knockout, IFN-α/βR^−/−^) have been found to be susceptible to wild-type SUDV (Brannan et al., 2015). Specifically, IFN-α/βR^−/−^ mice challenged i.p. with a dose of 10^3^ pfu of SUDV Boneface variant (SUDV/Bon) subsequently exhibited ~25% weight loss, which peaked at 7–9 dpi**.** Maximum lethality was observed in the 10^3^ pfu challenge group, with a 63% survival, and mean time to death was approximately 11 days. Histological analyses of liver and spleen samples of the SUDV-infected mice on day 3 showed the development of hepatocellular degeneration and necrosis.

## GUINEA PIG MODEL

### Ebola virus (EBOV)

Guinea pigs occupy a prominent role in filovirus research and are widely used for studying filoviral hemorrhagic fevers (Bowen et al., 1977; Cross et al., 2015a; Wong et al., 2015a, b, c). However, guinea pigs infected with WT filoviruses result in only transient illness (Ryabchikova et al., 1996; Simpson et al., 1968). Therefore, EBOV was serially passaged in the livers and spleens of guinea pigs until the adapted virus (GPA-EBOV) caused uniform lethality, with animals succumbing to death at 7–9 dpi (Simpson et al., 1968). At a dose of 103.8 pfu, GPA-EBOV-infected animals showed fibrin deposition and thrombocytopenia during the late stages of infection (Connolly et al., 1999), as well as high viral titers in the spleen, liver, adrenal gland, and lungs. Viremia was observed in guinea pigs 2 days after inoculation, which peaked on day 7 (>10^4^ pfu/mL) (Connolly et al., 1999; Subbotina et al., 2010).

Guinea pigs infected with GPA-EBOV showed drastic histopathological changes in Kupffer cells (especially virus replication), death of hepatocytes, and destruction of the sinusoid wall (Connolly et al., 1999), with the spleen and lymph nodes demonstrating lymphoid necrosis along with neutrophilia and lymphopenia (Connolly et al., 1999; Subbotina et al., 2010). Remarkably, lymphocyte bystander apoptosis was not prominent in these animals (Bradfute et al., 2007; Bray et al., 1998; Connolly et al., 1999). While the guinea pig model recapitulates some aspects of human filoviral disease, its higher unit cost, need for more specialized housing/husbandry requirements, and the lack of reagents to characterize aspects of innate/adaptive immune responses (such as T-cell immunity or cytokine levels) mean that it is more suited as a secondary animal model for confirming experimental results and trends from mice studies.

Adaptation of MARV (isolates Ci67 and Musoke) in guinea pigs from previous research resulted in their deaths on days 7–9, with body temperatures recorded at 41.1 °C (Simpson et al., 1968) and clinical symptoms such as bloated face and loss of appetite and weight evident. Cross et al. (2015b) evaluated and compared pathogenicity in outbred guinea pigs with GPA-RAVV_UTMB_ and GPA-MARV infections to better understand the disease pathogenesis of MARV infection in the guinea pig model. Results demonstrated prolonged clotting times (PT and APTT) associated with an increase in plasminogen activator inhibitor 1 and von Willebrand factor activity in GPA-RAVV and GPA-MARV-Angola-infected animals. In addition, circulating protein C activity and tissue factor concentrations also decreased during the time of death.

### Marburg virus (MARV)

Guinea pigs infected with approximately 5×10^3^ pfu of GPA-MARV-Angola showed remarkable differences in tissue factor kinetics (Cross et al., 2015b). In these animals, prolonged clotting times (PT and APTT) were associated with an increase in plasminogen activator inhibitor 1 and von Willebrand factor activity. Circulating protein C activity and tissue factor concentrations were also decreased at death. Thus, these proteins likely played a significant role in coagulopathy. Furthermore, disturbance of the fibrinolysis pathway was implicated by the depression in the metabolism of bradykinin, prekallikrein, and thrombin-activated fibrinolysis inhibitor (TAFI). Interestingly, GPA-RAVV-infected animals displayed increased TAFI concentrations at 3 dpi, whereas GPA-MARV-Angola-infected animals showed a decrease in TAFI concentrations at late stage infection. Inflammatory mediators such as TNF-a, IL-6, nitric oxide, and HMGB-1 were also elevated during the late stages of infection.

### Sudan virus (SUDV)

Wong et al. (2015a) developed and characterized a guinea pig-adapted SUDV variant based on the Boneface variant through serial passaging of WT-SUDV in the livers and spleens, and showed that the adapted virus caused uniform lethality in guinea pigs. The guinea pig-adapted SUDV variant (SUDV-GA) caused LD_50_ to animals at a dose of 5.3×10^-2^ 50% tissue culture infective doses (TCID_50_). Animals infected with SUDV-GA showed high viremia and died between 9–14 dpi. Several hallmarks of SUDV infection were observed, such as lymphadenopathy, elevated liver enzyme activities, and coagulation abnormalities. Histopathology and immunohistochemistry findings indicated that the SUDV-GA antigen was present in the livers and spleens of infected animals.

## SYRIAN HAMSTER MODEL

### Ebola virus (EBOV)

A Syrian golden hamster model for EBOV was developed by inoculating 6-week-old hamsters i.p. with 10^3^ focus forming units (ffu) of MA-EBOV (Ebihara et al., 2013; Zivcec et al., 2011). Hamsters i.p. inoculated with MA-EBOV (Bray et al., 1998) showed signs of disease, such as ruffled fur and decreased activity beginning at 3 dpi, and all animals died by days 4–5, with correspondingly high virus titers in the spleen, liver, kidneys, heart, lungs, and brain, indicating systemic spread of the virus (Ebihara et al., 2013). 

Viremia was detected beginning on day 2 and reached ~10^8^ pfu/mL by day 4, with infected hamsters showing rapid and increased distribution of the viral antigen in the spleen and liver (Ebihara et al., 2013). In the spleen, macrophages and marginal reticular-like cells in the red pulp and marginal zone showed higher virus replication. In the liver, Kupffer cells were the first target cells for MA-EBOV, which then spread to hepatocytes starting at 2 dpi. Importantly, coagulopathy was observed in the MA-EBOV-infected Syrian hamsters, but was absent in other rodent models. In addition, thrombocytopenia and decreased protein C concentrations were also noted in the infected hamsters. Using in-house primer-probe sets, Ebihara et al. (2013) also showed that MA-EBOV infection induced the expression of cytokine/chemokine genes, including IL-1b, IL-6, TNF-a, IL-12p35, IP-10, and IL-10, in the spleen and liver, indicating an uncontrolled immune response. Due to the presence of rash and induction of cytokines/chemokines, the Syrian hamster more closely recapitulates EBOV disease compared with guinea pigs; however, the higher unit costs, need for specialized housing/husbandry equipment, and lack of commercial reagents for studying innate and cell-mediated immune responses means that this model is still not widely used in filovirus research.

### Marburg virus (MARV)

Marzi et al. (2016) recently developed a novel Syrian golden hamster model for MARV infection using a hamster-adapted MARV variant Angola (HA-MARV). This model provides new insight on virus pathogenesis in hamsters, and moreover closely resembles MARV infection in humans and NHPs, including hemorrhagic manifestations and coagulation abnormalities. In this study, hamsters were infected i.p. with 10^–1^– 10^3^ pfu of HA-MARV, leading to significant weight loss and disease surrender between 7–11 dpi. The HA-MARV infected hamsters displayed an increase in body temperature at 6 dpi and drop in temperature during the end stage of the disease. In contrast, the mock-infected animals (WT-MARV) did not show any changes in body temperature or weight, suggesting that infection with WT-MARV failed to induce the disease in hamsters. Maculopapular rashes along with visible petechial hemorrhages on the face and abdomen were noted in the HA-MARV-infected hamsters on 7 dpi. Hemorrhage was also identified in the footpads and joints, as well as in the kidneys. Coagulation parameters in HA-MARV-infected hamsters such as PT, APTT, and thrombin increased with delay in blood clot formation during the infection. However, the animals infected with WT-MARV did not exhibit these coagulation abnormalities. The HA-MARV replication was found to be robust, with viremia peaking at 10^8^ TCID_50_/mL in the blood, 10^7^ TCID_50_/mL in the liver, 10^6^ TCID_50_/mL in the spleen, and 10^5^ TCID_50_/mL in the mesenteric lymph nodes by 7–8 dpi. Dysregulated immune responses were also observed in hamsters infected with HA-MARV; for example, pro-inflammatory chemokines MIP-1α and IP-10, as well as type I interferon responses, were elevated when compared to WT-MARV-infected animals.

## FERRET MODEL

### Ebola virus (EBOV)

The susceptibility of domestic ferrets (*Mustela putorius furo*) to EBOV has been studied recently. Cross et al. (2016) demonstrated that ferrets infected intranasally (i.n.) with the EBOV Kikwit variant at 10^3^ pfu, showed uncontrolled viral replication, abnormalities in white blood cell counts, and multiple-organ failure. Initial signs of the disease in the EBOV-infected ferrets were first observed at 3 dpi, followed by rapid-onset hypothermia and weight loss on day 4. Remarkably, ferrets died between 6–9 dpi. Necropsy inspection revealed pathological features of hemorrhagic fever, including petechial rashes on the skin surface, reticulated pallor of the liver, and mottled splenomegaly. All ferrets showed progressive neutrophilia and lymphocytopenia beginning on 4 dpi. Vascular leakage was also noted with hypoalbuminemia and hypoproteinemia, and increasing levels of circulating proinflammatory markers TNF-α and nitric oxide were also recorded on day 4. 

Kozak et al. (2016) also reported on a ferret model for EBOV. Animals were infected with 200 TCID_50_ via the i.m or i.n route, and showed peak viremia of 10^7^ GEQ/mL or 10^9^ TCID_50_/mL by 5 or 6 dpi, with evidence of sporadic viral shedding from the oral, nasal, and rectal cavities. Notably, weight loss was less pronounced in animals infected with EBOV compared with BDBV-infected animals from the same study. Biochemical analyses further demonstrated increases in concentrations/ activities of ALT, ALP, and BIL, suggesting liver damage, and increases in BUN and CRE, indicating elevated renal problems. Prolonged APTT and thrombin time (TT), along with an increase in fibrinogen levels, were also noted, suggesting that ferrets recapitulated disseminated intravascular coagulation. Interestingly, EBOV-infected ferrets displayed decreased levels of serum albumin after EBOV challenge, which might be the cause of disease-induced edema.

The results from the above studies demonstrate that ferrets are highly susceptible to EBOV infection and the pathological parameters observed during disease pathogenesis closely resemble those found in humans. 

### Sudan virus (SUDV)

Kroeker et al. (2017b) demonstrated that ferrets are susceptible to wild-type SUDV infections. Ferrets inoculated with WT-SUDV via i.m and i.n experienced viremia, organ dysfunction, viral shedding, and eventual death. A decrease in white blood cell counts, increase in fibrinogen, and decrease in platelets and PT%, which are indicative symptoms of disseminated intravascular coagulopathy, were observed further during disease progression. Cross et al. (2016) also described a lethal model of SUDV with the domestic ferret. Ferrets were challenged i.n. with 10^3^ pfu of SUDV, with initial signs of the disease observed at 3 dpi and rapid-onset hypothermia and weight loss detected by day 4 for SUDV-infected ferrets. Clinical signs included diarrhea, dehydration, nasal and ocular discharge, labored breathing, hunched posture, and altered gait, with death occurring at 6–9 dpi. Hematoxylin-eosin staining revealed that the most significant lesions in the infected ferrets were lymphohistiocytic and neutrophilic necrotizing hepatitis and necrotizing splenitis.

### Bundibugyo virus (BDBV)

Kozak et al. (2016) challenged ferrets with 159 TCID_50_ of BDBV via the i.m. route, and observed viremia at 4 dpi and uniform lethality at 8 dpi. In all BDBV-infected animals, significant increases in coagulation parameters were observed, including in APTT, TT, and PT. Hematological analysis revealed a decrease in white blood cell counts, lymphocytes, and platelets in ferrets after infection. Petechial rashes were also observed during the disease.

## NON-HUMAN PRIMATE(NHP)MODELS

### Ebola virus (EBOV)

Many reports have demonstrated African green monkeys (*Chlorocebus aethiops*), cynomolgus macaques (*Macaca fascicularis*), and rhesus macaques (*Macaca mulatta*) as excellent models for EBOV infection (Bowen et al., 1978; Davis et al., 1997; Ellis et al., 1978; Fisher-Hoch et al., 1992; Geisbert et al., 2003a, b, c, d; Ignatiev et al., 2000; Jaax et al., 1996; Jahrling et al., 1996; Johnson et al., 1995; Ryabchikova et al., 1999; Wong et al., 2016). Following EBOV infection, NHPs became febrile (above 40 °C) at 3 dpi, with pyrexia and temperature decrease exhibited throughout the course of the disease, followed by death within 5–8 dpi (Baskerville et al., 1978; Bowen et al., 1978; Ellis et al., 1978; Fisher-Hoch et al., 1985; Luchko et al., 1995). Viremia has been observed at 3 dpi with titers reaching 10^6.5^−10^7^ pfu/mL on days 4–5 (Bowen et al., 1978; Fisher-Hoch et al., 1992; Jahrling et al., 1996). Compared with other NHP species, cynomolgus and rhesus macaques are the best available animal models due to their susceptibility to EBOV infections (Bente et al., 2009), thus providing a stringent test for any candidate medical countermeasure. In addition, African green monkeys and baboons infected with EBOV show impairment of the coagulation system. For example, fibrin thrombosis was localized to all visceral organs in African green monkeys, whereas hemorrhage was most prominent in the visceral organs such as the liver and spleen in baboons (Ignatiev et al., 2000; Ryabchikova et al., 1999).

Currently, macaques well recapitulate pathological features of fatal filovirus disease observed in humans, including high viremia, coagulation abnormalities such as decreased platelet levels and increased blood clotting times, and aberrant proinflammatory cytokine responses, including the release of IL-6 and MCP-1 (Geisbert et al., 2003a, 2015). Studies in macaques have also demonstrated that doses of EBOV as low as 2−15 pfu administered by different challenge routes can produce lethal filovirus infection (Sullivan et al., 2000, 2003). Ebola virus disease (EVD) signs in macaques initially occur at 3−5 days after exposure, with symptoms including fever and malaise, followed by anorexia, depression, lethargy, diarrhea, vomiting, and maculopapular rash. Hemorrhagic manifestations have also been identified, and include petechiae, ecchymosis, and bruising, hemorrhage at venipuncture sites, epistaxis, hematochezia, and hematuria. In addition, neutrophilia, lymphopenia, thrombocytopenia, and early monocytosis indicate abnormalities in complete blood count parameters. 

Although airborne transmission is not considered a significant route of human infection (Osterholm et al., 2015), aerosolized viruses have caused lethal disease in experimentally-infected NHPs. Previous study showed that control rhesus macaques, located 3 m from experimental rhesus macaques inoculated i.m. with EBOV, became infected (Jaax et al., 1995), with antigen staining patterns of pulmonary specimens implicating aerosol infection. Conversely, other research has shown no transmission between rhesus macaques infected i.m. with EBOV and uninfected rhesus macaques when housed adjacent to each other (Alimonti et al., 2014). However, virus transmission from pigs infected oro-nasally with EBOV to naïve NHPs without direct contact was shown to be possible, with the infected NHPs exhibiting extensive lung damage (Weingartl et al., 2012). Mucosal exposure as a source of infection in rhesus macaques (through conjunctival and oral routes), required higher doses (5.2 log_10_ of EBOV Mayinga isolate) compared to parenteral routes; however, doses of 10 pfu by oral or conjunctival routes did not result in any clinical disease (Mire et al., 2013). Thus, despite the above studies, an aerosol model of EBOV infection in NHPs consistently causing 100% lethality has not yet been established.

### Marburg virus (MARV)

NHPs are susceptible to MARV infections and have been extensively used to study its pathogenesis. Fernando et al. (2015) reported that cynomolgus macaques showed severe disease against Marburg Angola compared with other MARV strains, with high fever, anorexia, and lymphopenia, followed by death (mean time after infection of 6.7 days). Peak viremia reached 10^4^–10^7^ TCID_50_/mL by 4 dpi. Cynomolgus macaques showed febrile illness, anorexia, diarrhea, skin rash, and hemorrhagic manifestations at 2–6 dpi following high dose (10^3^ pfu) infection by MARV Angola (Alves et al., 2010; Geisbert et al., 2007; Hensley et al., 2011; Simpson et al., 1968; Simpson, 1969; Murphy et al., 1971), with a sudden decrease in body temperature followed by death at 6–13 dpi. Lymphocytosis was observed at the beginning of the illness (Geisbert et al., 2007; Gonchar et al., 1991; Johnson et al., 1996; Simpson et al., 1968; Simpson, 1969; Spiridonov et al., 1992), whereas thrombocytopenia and leukocytosis with increased neutrophilia were observed 5–6 dpi (Hensley et al., 2011). The infection was also observed to spread to the liver, adrenal glands, and finally to endothelial cells in a variety of organ tissues (Hensley et al., 2011). Viremia occurred on day 3 in cynomolgus macaques and African green monkeys, reaching peak titers of 10^7^−10^8^ pfu/mL at 8 dpi (Hensley et al., 2011).

### Sudan virus (SUDV)

SUDV infection has been shown to cause death in the NHP model (rhesus macaques) 7−10 dpi (Thi et al., 2016). Specifically, adult rhesus macaques inoculated i.m. with 10^3^ pfu of SUDV Gulu succumbed to infection after 7–10 days, with viremia peaking at 10^8^ pfu/mL at 7 dpi, accompanied by liver and renal dysfunction as evidenced by increased ALT, GGT, BUN, and CRE levels. Control and treated animals that succumbed to SUDV infection showed lesions (observed by hematoxylin and eosin staining) in tissues. Significant lesions included splenic lymphoid depletion with tangible body macrophages and fragmented nuclear debris and enlargement of splenic red pulp with fibrin, multifocal necrotizing hepatitis with sinusoidal leukocytosis, and mild interstitial pneumonia. Furthermore, depression, anorexia, petechial rash, and hemorrhage were observed in the animals infected with SUDV.

### Bundibugyo virus (BDBV)

Cynomolgus macaques have been used for studying the pathogenesis of BDBV infection (Mire et al., 2013), in which macaques were challenged i.m. with 10^3^ pfu of BDBV. Animals were monitored for clinical signs of illness over the course of 28 days. Clinical scores were recorded each day post-challenge for each animal using a scoring system based on dyspnea, depression, recumbency, and rash. No antibody titers (IgG) for the control group were observed against BDBV. The signs of disease in response to BDBV infection were more dramatic for the control animals. Signs observed between day 0−28 after BDBV challenge included fever, anorexia, depression, rectorrhagia, lymphopenia, and thrombocytopenia. Serum concentrations of alkaline phosphatase (ALP), aspartate aminotransferase (AST), blood urea nitrogen (BUN), and gamma glutamyltransferase (GGT) were elevated, and by days 10−11 all animals had died.

### Tai Forest virus (TAFV)

TAFV may cause severe disease and death in NHPs, as evidenced by the severe infection of one person after coming into contact with a dead chimpanzee, although the human patient ultimately survived (Guenno et al., 1995). To date, there have been no other reported infections, and thus this virus has not been the subject of extensive studies and there is a lack of animal models.

## SUMMARY

Animal models are important for the study of filovirus pathogenesis, with the goal of testing the effectiveness of specific antivirals and vaccines. A variety of animal species are susceptible to WT and host-adapted filoviruses, and are thus used to test the efficacy of anti-filovirus products during non-outbreak situations. The Food and Drug Administration’s “two-animal rule” states that the efficacy of a candidate product should be tested in “more than one animal species expected to react with a response predictive for humans” (FDA, 2017). Therefore, any vaccine or antiviral must demonstrate safety and efficacy in at least two animal models (a smaller animal model in addition to a NHP model) to have any hope of being licensed. 

The most important aspects of filovirus infection are summarized in Table 3. Parameters such as lymphopenia, liver damage, thrombocytopenia, coagulopathy, cytokine storm, rash, and hemorrhage are the chief hallmarks of filoviral diseases. Due to their advantages in terms of handling, cost, and access, mice and guinea pigs often serve as primary and secondary animal models for preliminary experiments and diverse genetic studies (such as knockout mice); however, as viruses can adapt to hosts and each host cannot recapitulate all hallmarks of filovirus diseases, mice and guinea pigs have limited translatability and predictability to human filoviral disease and thus are mostly used to screen potentially efficacious compounds. Currently, NHPs are the only animal species available that can recapitulate all stated aspects and thus are considered as the gold standard. Ferrets are the newest animal model to gain attention in filovirus research (Cross et al., 2016; Kozak et al., 2016; Kroeker et al., 2017b). In addition to being small, ferrets can accurately recapitulate human filoviral diseases, including coagulopathy. Syrian golden hamsters can also recapitulate disease that closely mimics that in humans and NHPs, including coagulopathy. However, this model suffers from the need for host-adapted viruses and the lack of widespread reagents for studying immune responses. Therefore, ferrets and NHPs are likely the two best animal models at present for the testing of anti-filovirus compounds.

**Table 3:** Clinical manifestations in different animal models of filovirus infections

	Immuno-competent mouse	Immuno-comprimised mouse	Guinea pig	Syrian hamster	Ferret	NHP	Human
Host-adapted virus	Yes	Yes	Yes	Yes	No*	No*	No*
Lymphopenia	Yes	Yes	Yes	Yes	Yes	Yes	Yes
Liver damage	Yes	Yes	Yes	Yes	Yes	Yes	Yes
Thrombocytopenia	Yes	Yes	Yes	Yes	Yes	Yes	Yes
Coagulopathy	No	Unknown	Yes	Yes	Yes	Yes	Yes
Cytokine storm	Yes	Yes	Unknown	Yes	Unknown	Yes	Yes
Rash	No	No	No	Yes	Yes	Yes	Yes
Hemorrhage signs	No	Yes	Unknown	Yes	Yes	Yes	Yes

NHP: Non-human primates; No*: Wild-type nonadapted viruses.

While this review has covered the different animal models available for each filovirus (Table 1), it is important to note that models against some viruses are more developed than others. There is an abundance of EBOV animal models, but very limited models available against TAFV. In addition, only ferrets have been used as a small animal model for BDBV. Immediate future goals should be the development and characterization of rodent animal models for the lesser known filoviruses. With experience gained from previous studies and well-established methods, the goal of establishing the necessary animal models for each known filovirus is attainable.
